# High Resistance of Vector of West Nile Virus, *Culex pipiens* Linnaeus (Diptera: Culicidae) to Different Insecticides Recommended by WHO in Northern Iran

**Published:** 2018-03-18

**Authors:** Fatemeh Ghorbani, Hassan Vatandoost, Ahmad Ali Hanafi-Bojd, Mohammad Reza Abai, Hassan Nikookar, Ahmad Ali Enayati

**Affiliations:** 1 Department of Medical Entomology and Vector Control, School of Public Health, Tehran University of Medical Sciences, Tehran, Iran; 2 Department of Environmental Chemical Pollutants and Pesticides, Institute for Environmental Research, Tehran University of Medical Sciences, Tehran, Iran; 3 Department of Medical Entomology and Vector Control, Student Research Committee, Health Sciences Research Center, School of Public Health, Mazandaran University of Medical Sciences, Sari, Iran; 4 Department of Medical Entomology and Vector Control, School of Public Health, Mazandaran University of Medical Sciences, Sari, Iran

**Keywords:** *Culex pipiens*, Insecticide resistance, Temephos, Propoxur, Deltamethrin

## Abstract

**Background:**

*Culex pipiens* is a mosquito species distributed in different parts of Iran. It is known as vector of some diseases as well as a nuisance insect. A successful control campaign needs to study the biology, ecology and susceptibility status of the target pest. The aim of this study was to investigate the susceptibility status of *Culex pipiens* to some insecticides recommended by WHO in north of Iran.

**Methods:**

Larvae were collected from different breeding places in Sari County by standard dipping method. They were reared to adult stage in an under standard condition. World Health Organization (WHO) standard test kits and the diagnostic dose of 12 imagicides were used for adults, while two larvicides were used against larvae.

**Results:**

The least and highest mortality rates after exposure to insecticides were 4.7% (Propoxur 0.1%) and 76.4% (Etofenprox 0.5%), respectively. Larvae showed high resistance to temephos (19.19%) and malathion (54.63%). *Culex pipiens* was found to be resistant to all used insecticides/larvicides at diagnostic dose.

**Conclusion:**

Intensive use of pesticides against agriculture and urban pests increased the resistance level of this species to different insecticides, although currently there is no control program against it. Study on the mechanisms of resistant in this species to different insecticides is recommended.

## Introduction

More than 3,500 species of mosquito have been identified so far in the world, but few of them are vectors of the disease to humans. Medical importance and the geographical distribution of mosquitoes vary in different regions (1). *Wuchereria bancrofti* and *Brugia malayi* parasites are distributed in tropical and subtropical Africa, Asia, Australia and the Pacific and transmitted by *Culex pipiens* Linnaeus. In addition, Arboviral diseases such as West Nile, Sindbis, Japanese encephalitis, Western equine encephalitis, Rift valley fever, Tahyna and Oropouche transmitted by *Cx. pipiens*. The transmission of birds malaria caused by *Plasmodium gallinacum* is also proven by this species (2). Among these diseases, West Nile viral disease transmitted by mosquitoes has been detected serologically in humans from Guilan, Khorasan, Khuzistan, Sistan and Baluchestan and Isfahan Provinces of Iran (3–5). Dog heart worm parasites, *Dirofilaria immitis* and *Dirofilaria repens* also reported from human, dog and mosquito in the country (6, 7).

Due to high adaptability and potential of reproduction, *Cx. pipiens* breeds in a variety of larval habitats from pits containing waste water, polluted water to holes and dishes of the clean water (2). The most basic control method of mosquitoes is chemical insecticides, although some biological methods are used against malaria vectors. Control programs should be regularly carried out using the updated data on the susceptibility of mosquitoes to the insecticides. Invasive and irrational use of pesticides has led to the development of resistance to insecticides in insects. Hence, the survey on resistance of mosquitoes to insecticides ensures us to do an effective operation. In studies conducted around the world, *Cx. pipiens* has been shown resistant to some pesticides (8–12). *Culex pipiens* and *Cx. molestus* have shown resistance to most of organophosphates in Iran, Iraq, and Egypt and to carbamates in Iraq and Kuwait. Overall, this mosquito has shown resistance to 36 pesticides in different countries (https://www.pesticideresistance.org).

Mazandaran Province is one of the main agricultural and horticultural poles of Iran and the largest consumer of pesticides (Unpublished data). According to a current study about 14,000 tons of agriculture pesticides, expressed in active ingredients (AI), were annually used in Iran during 2012–2014 (13). So far no study has been done on the susceptibility of *Cx. pipiens*, a common urban pest, in Sari County, north of Iran. This city is one of the most important tourist destinations in the country. Therefore, this study was aimed to test the effectiveness of the WHO recommended pesticides against the mosquito and to find the most effective compounds.

## Materials and Methods

### Study area

**Fig. 1.**
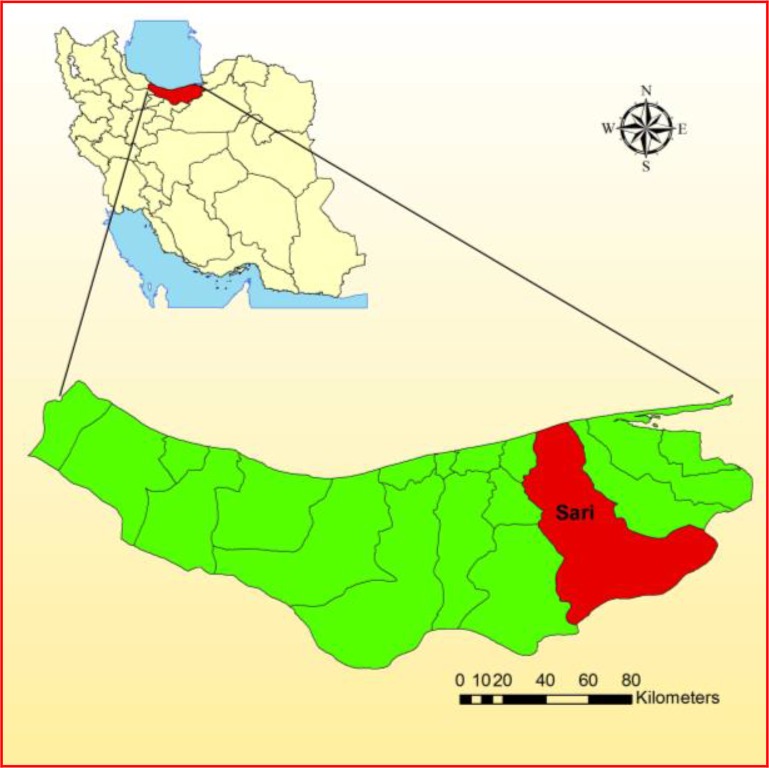
Geographical poistion of the study area in north of Iran

Sari, the provincial capital of Mazandaran Province is considered as an important and one of the most attractive and beautiful regions of northern Iran. It is located in 53.08 °E and 36.56 °N with an altitude of 40m above the sea level (Fig. 1). Agriculture and horticulture are the most important jobs in the region.

### Sampling

To find the larval habitats of choice for *Cx. pipiens*, sampling of different larval habitats in the city of Sari was carried out using standard dipping method. Physical characteristics and geographic coordinates of collection sites were recorded using a GPS device and registered in the relevant forms. The collected larvae were transferred to the laboratory, mounted and identified using morphological keys (14). From the end of June 2016 and after finding the best larval habitats for *Cx. pipiens*, sampling was started for susceptibility tests.

### Susceptibility tests for adult mosquitoes

According to World Health Organization guidelines (15), diagnostic dose of different pesticides were used against larvae and female adult mosquitoes. A total of 4 replicates representing 100 specimens were used for each pesticide and 50 for control. The following insecticides were used: DDT4%, Dieldrin 0.4%, Dieldrin 4%, Malathion 5%, Fenitrothion 1%, Propoxur 0.1%, Bendiocarb 0.1%, Etofenprox 0.5%, Cyfluthrin 0.15%, Permethrin 0.75%, Lambdacyhalothrin 0.05% and Deltamethrin 0.05% (15). Dose and exposure time for different insecticides was according to WHO procedures (16).

Tests were carried out in a room with 25–30 °C temperature and 65–75% RH. Two- to three-days old sugar fed female mosquitoes were used in 4 replicates of 25 mosquitoes for each insecticide at diagnostic dose and 2 replicates as control. The exposure time was 0.5 to 4 hours according to WHO procedure for *Culex* mosquitoes (16). All tested specimens were identified after the test using morphological key (14) and non-target species were excluded from the results.

### Susceptibility tests for larvae

Two larvicides, i.e. temephos (0.02mg/l) and malathion (1mg/l) were used at the diagnostic dose provided by WHO (17). Larvae were collected from the identified breeding sites for *Cx. pipiens* in the city, transferred to the insectary and after 24 hours recovery time, late 3^rd^ or early 4^th^ instars were used for the tests. WHO test procedure for mosquito larvae was used in this study (17). Mortality rate was recorded after 24h exposure time.

### Data analysis

In the event of control mortality of less than 5% the results of the test were considered to be correct, 5–20% the results were corrected using Abbotts' formula and more than 20% the tests were discarded and repeated by new specimens. Mortality of the test between 98–100% was considered as susceptible, 90–97% as candidate of resistance that should be confirmed using specific methods, and less than 90% was considered as resistant (15).

## Results

**Fig. 2.**
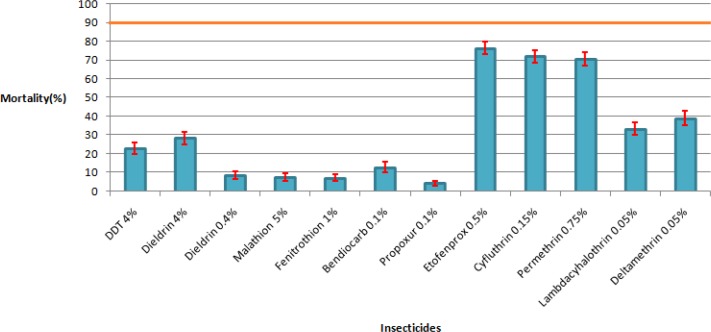
Mortality rate of *Culex pipiens* collected from Sari County, North of Iran using diagnostic dose of imagicides (red line indicate the resistant level)

*Culex pipiens* from Sari County, Northern Iran, was resistant to all used insecticides and larvicides in the diagnostic dose recommended by WHO. This mosquito showed high resistance level to the insecticides from four main groups of pesticides, i.e. organo-chlorine, organophosphate, carbamate and pyrethroids (Table 1, Fig. 2). However, the resistance level was lower to pyrethroids compared to other insecticides. The highest and the least mortality rates were found to Etofenprox and Propoxur with a mortality rate of 76.47 and 4.25%, respectively. This mosquito species was resistant to both larvicides (Table 1), although malathion killed more larvae (54.63%) compared to temephos (19.19%). Overall, the mortality rate was the highest to organophosphates and carbamates, followed by organochlorines and pyrethroids.

**Table 1.** Response of *Culex pipiens* collected from Sari County, Northern Iran, 2016 to different insecticides/larvicides

**Insecticide/Larvicide**	**Exposure time (Minute)**	**Mortality (%)**		

		**Mean**	**SD**	**Control**
DDT 4%	240	22.98	3.2	4.1
Dieldrin 4%	60	28.57	3.3	0
Dieldrin 0.4%	60	8.69	2	0
Malathion 5%	60	7.52	1.9	2.22
Fenitrothion 1%	120	7.31	2	2.2
Bendiocarb 0.1%	60	12.94	2.6	0
Propoxur 0.1%	120	4.25	1.4	0
Etofenprox 0.5%	60	76.47	3.2	0
Cyfluthrin 0.15%	60	72.09	3.4	0
Permethrin 0.75%	60	70.73	3.5	2.2
Lambdacyhalothrin 0.05%	30	33.33	3.4	2.2
Deltamethrin 0.05%	30	39.08	3.7	2.2
Malathion (1ppm)	1440	54.63	3.6	0
Temephos (0.02ppm)	1440	19.19	2.8	0

## Discussion

Although organophosphates and carbamates have the same mechanism of action, there are reports of cross-resistance to pyrethroids and organophosphates in *Culex quinquefasciatus* Say (18, 19). Organophosphate insecticides have an extensive use in agriculture and horticulture in the study area. So, although there is no program for mosquito control in the area, continuous exposure of the insects to different insecticides, makes them resistant to other groups as well.

The same studies in Iran were recently carried out to find the susceptibility status of *Cx. pipiens.* The results showed that this species was resistant to DDT 4% in southeastern city of Chabahar (54.4% mortality), northwestern cities of Urmia (15.6%) and Ahar (23%) and the capital city Tehran (0%), (20–24). In other countries, such as China (11), Malaysia (10) and India (12), DDT resistant strains of *Cx. pipiens* and *Cx. quinquefasciatus* were reported. In natural conditions, *Cx. pipiens* breeds in sewage and water canals contaminated with different pesticides and other chemical pollutants. This continual exposure to pesticides acts as a natural selection pressure and increases the rate of resistant population. High resistance level to propoxur, bendiocarb and organophosphates in our study can be due to this reason. The Iranian Northern Province of Mazandaran has the most use of pesticides in the country (Unpublished data). Therefore, it is likely that all pests including *Cx. pipiens* develop resistance.

Mortality after exposure to pyrethroids in our study ranged between 33.33–76.47%. Other studies in Iran reported a range of 18–93% (20, 21–25), with the highest resistance rate in Tehran. Resistance to pyrethroids was also reported in this mosquito species from China and Saudi Arabia (11, 26). Larviciding may be considered as the main method for control of *Cx. pipiens* in urban areas. We found this species resistant to both larvicides. A study conducted in Tehran reported 8% mortality of this mosquito to temephos (27). This rate was 19.19 % in our survey. Esterases may be responsible for resistance to organophosphates (28). Studies are recommended to find the mechanism(s) of resistance to organophosphates, especially larvicides, in the study area.

## Conclusion

In conclusion, this is the first time that new diagnostic doses recommended by WHO are used against *Cx. pipiens*, so the results can be useful for other researchers. High resistance of this species to 14 insecticides/larvicides in Sari County, Northern Iran, reflects the situation of indiscriminate use of pesticides in the area. Although spraying is not done by the health system in the region, the widespread use of pesticides in household, agriculture and horticulture caused severe resistance to pesticides in *Cx. pipiens* Given the role of this mosquito in transmission of some important diseases, and annoyance caused by mosquito bites, judicious use of pesticides in all sectors including agriculture, horticulture, urban and public health pests is highly recommended. A comprehensive study on the mechanisms of resistance in *Cx. pipiens* to different insecticides is advised in Iran.
